# Supplementary figures and images for: Competition and fixation of cohorts of adaptive mutations under Fisher geometrical model
Source: PeerJ. 2016 Aug 2;4:e2256. doi: 10.7717/peerj.2256 (PMC4975028; doi:10.7717/peerj.2256)

Figure S1

$m = 3$

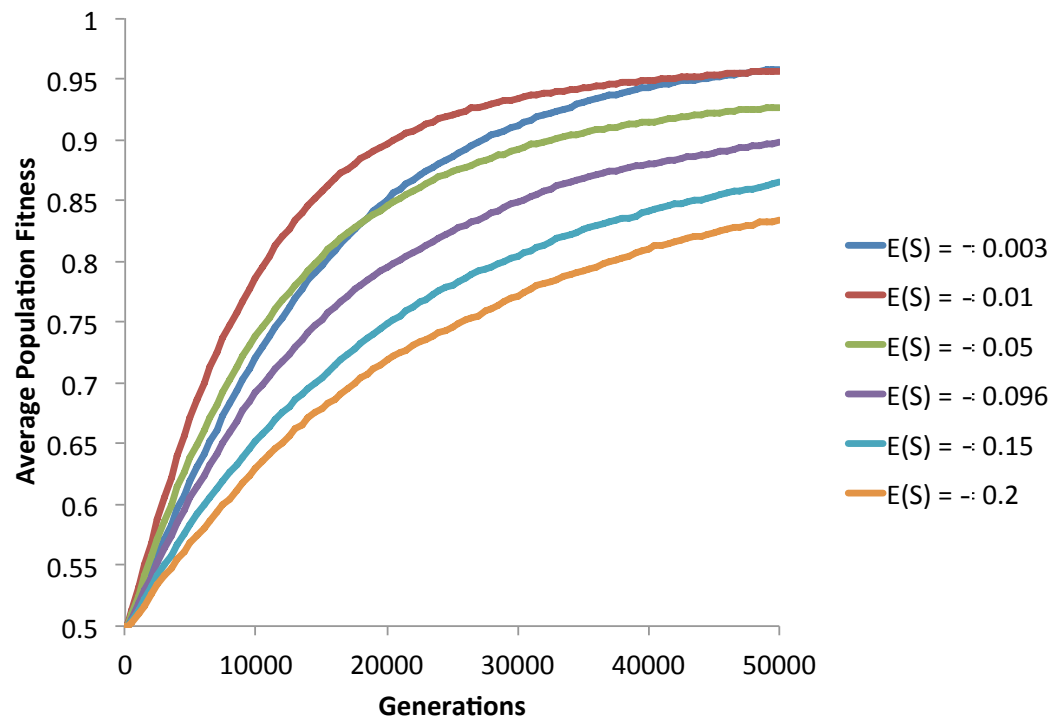

$m = 96$

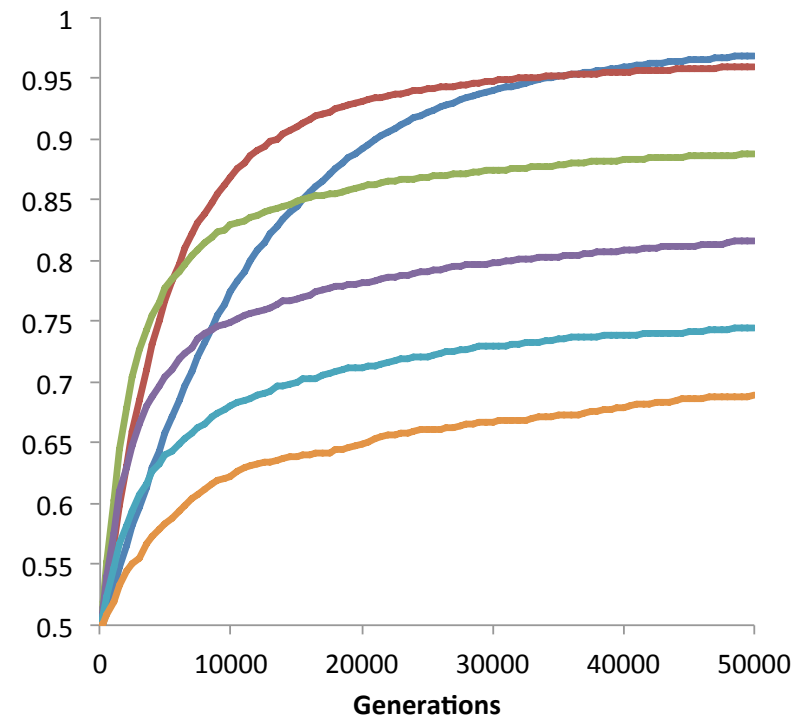

Supplement: Figure S1 — Dynamics are shown for high and low levels of pleiotropy (m = 96 and m = 3). Other parameters are as in Fig. 1. [file peerj-04-2256-s001.pdf]

Figure S2

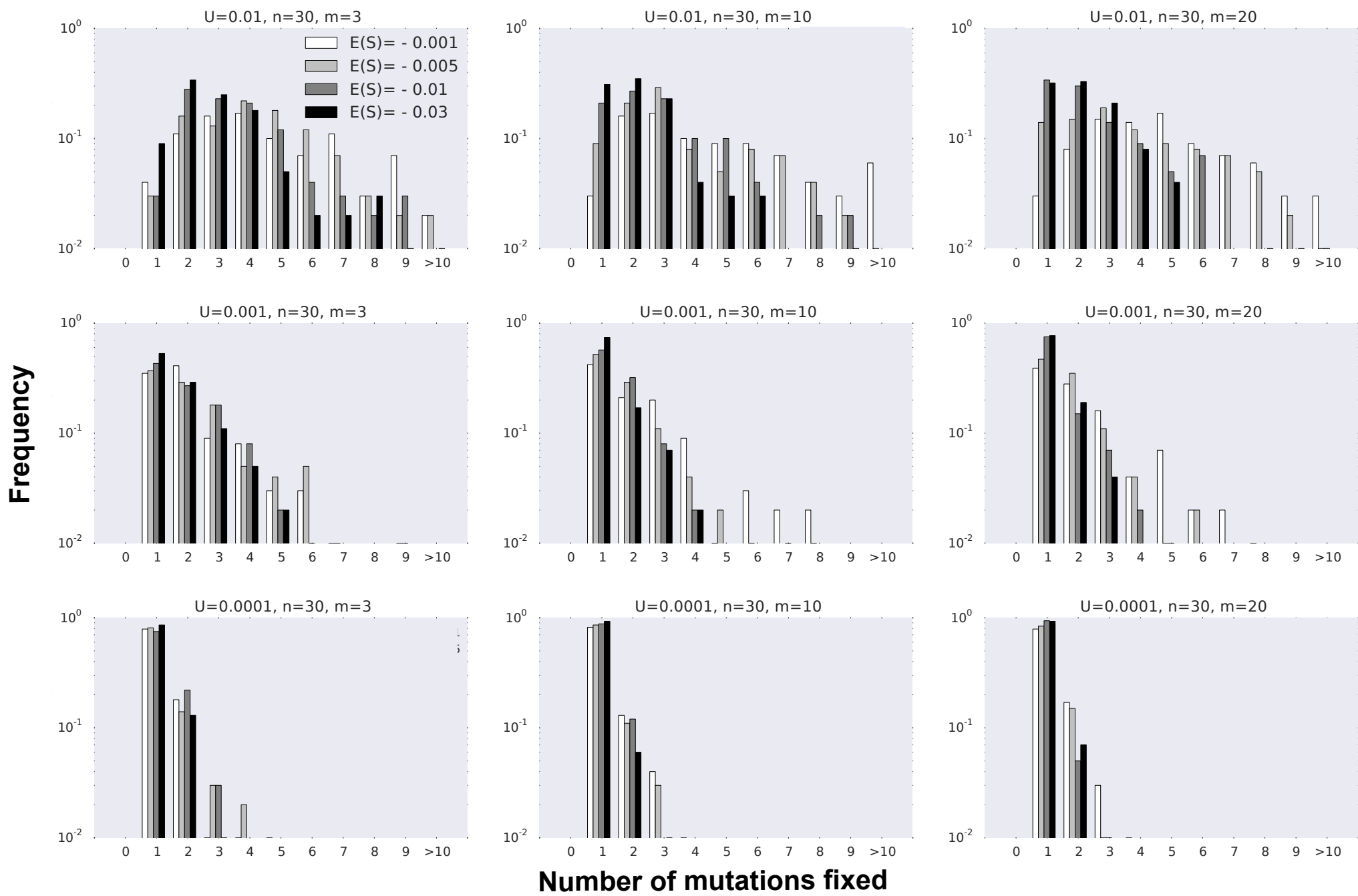

Supplement: Figure S2 — The complexity, n, is fixed at 30 while the pleiotropy, m, varies between 3, 10 and 20. Remaining parameters are as in Fig. 2. [file peerj-04-2256-s002.pdf]

Figure S3

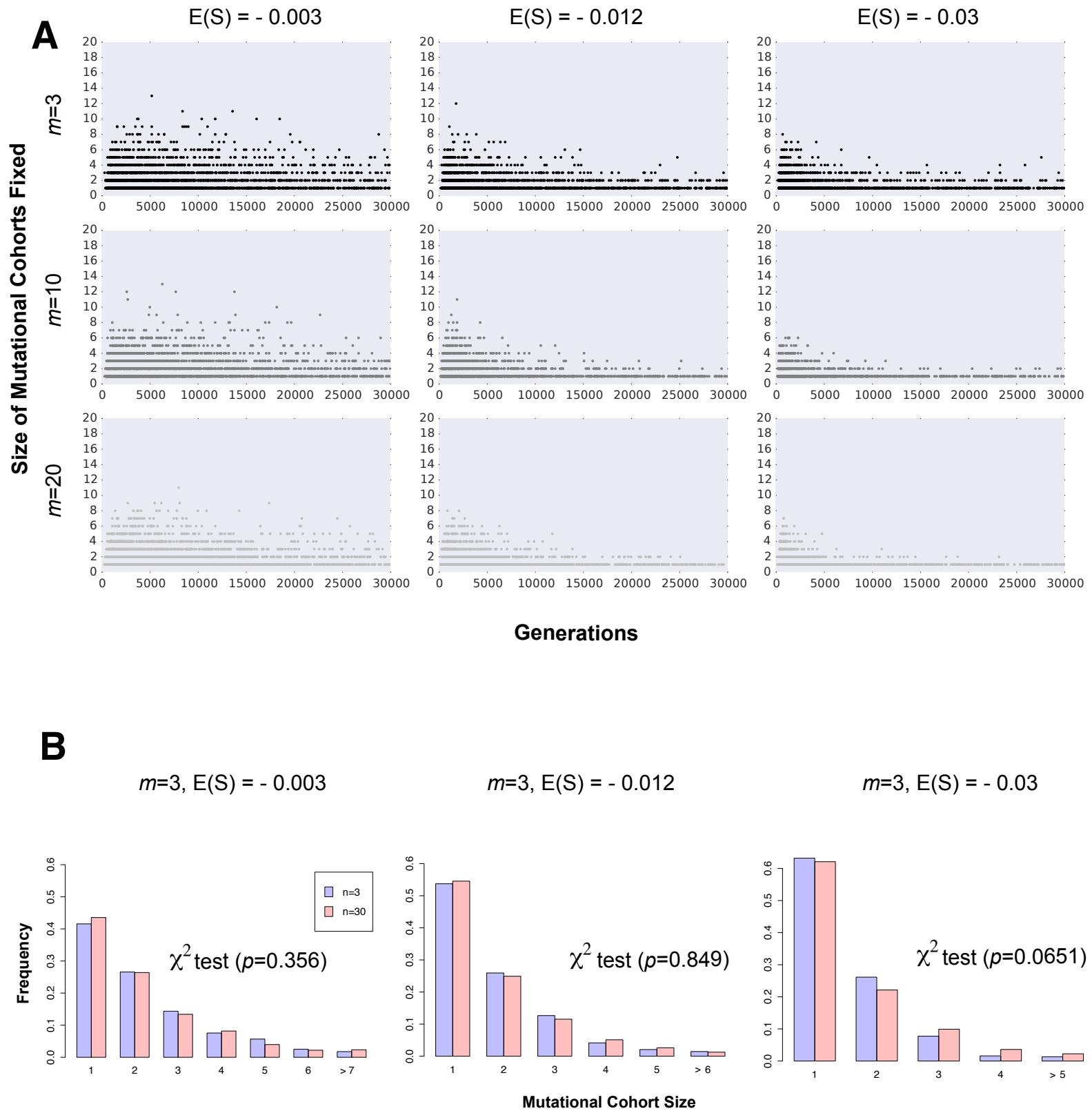

Supplement: Figure S3 — (A) The complexity, n, is fixed at 30, while the pleiotropy, m, varies between 3, 10 and 20. Remaining parameters are as in Fig. 3. (B) Histograms with frequencies of the number of mutations fixed in each cohort, across the 30,000 generations, for populations with full (n = 3, m = 3, blue distributions) or partial (n = 30, extitm = 3, red distributions) pleiotropy. Shown are the distributions for populations with different mean effect of mutations (E(S)). For each E(S), Chi-square (χ2) test for comparison of the distributions under partial or full pleiotropy is shown. [file peerj-04-2256-s003.pdf]

Figure S4

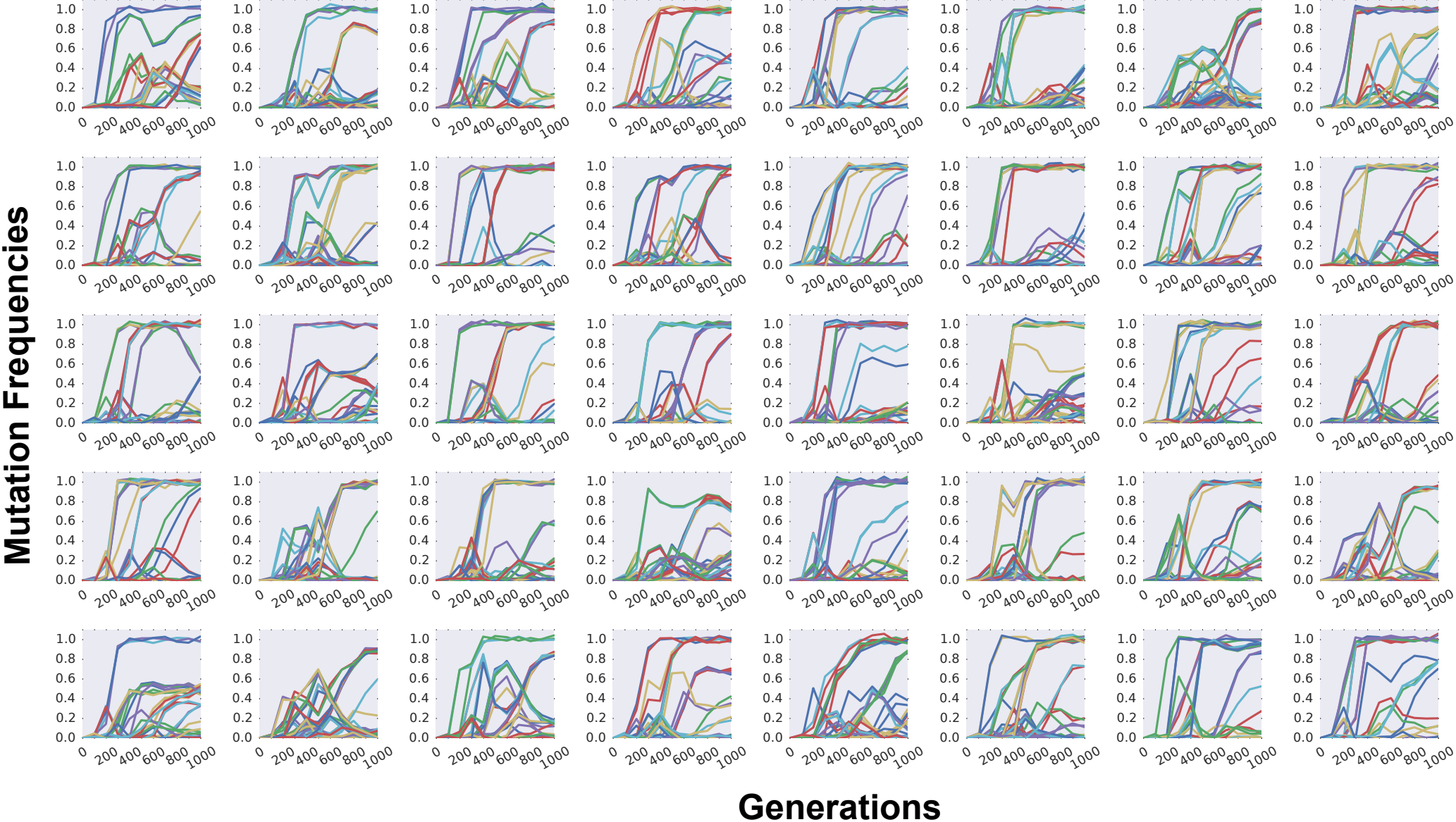

Supplement: Figure S4 — The diverse patterns of cohorts observed emerge from the dynamics of adaptation under FGM. Parameters are as in Fig. 4. [file peerj-04-2256-s004.pdf]

Figure S5

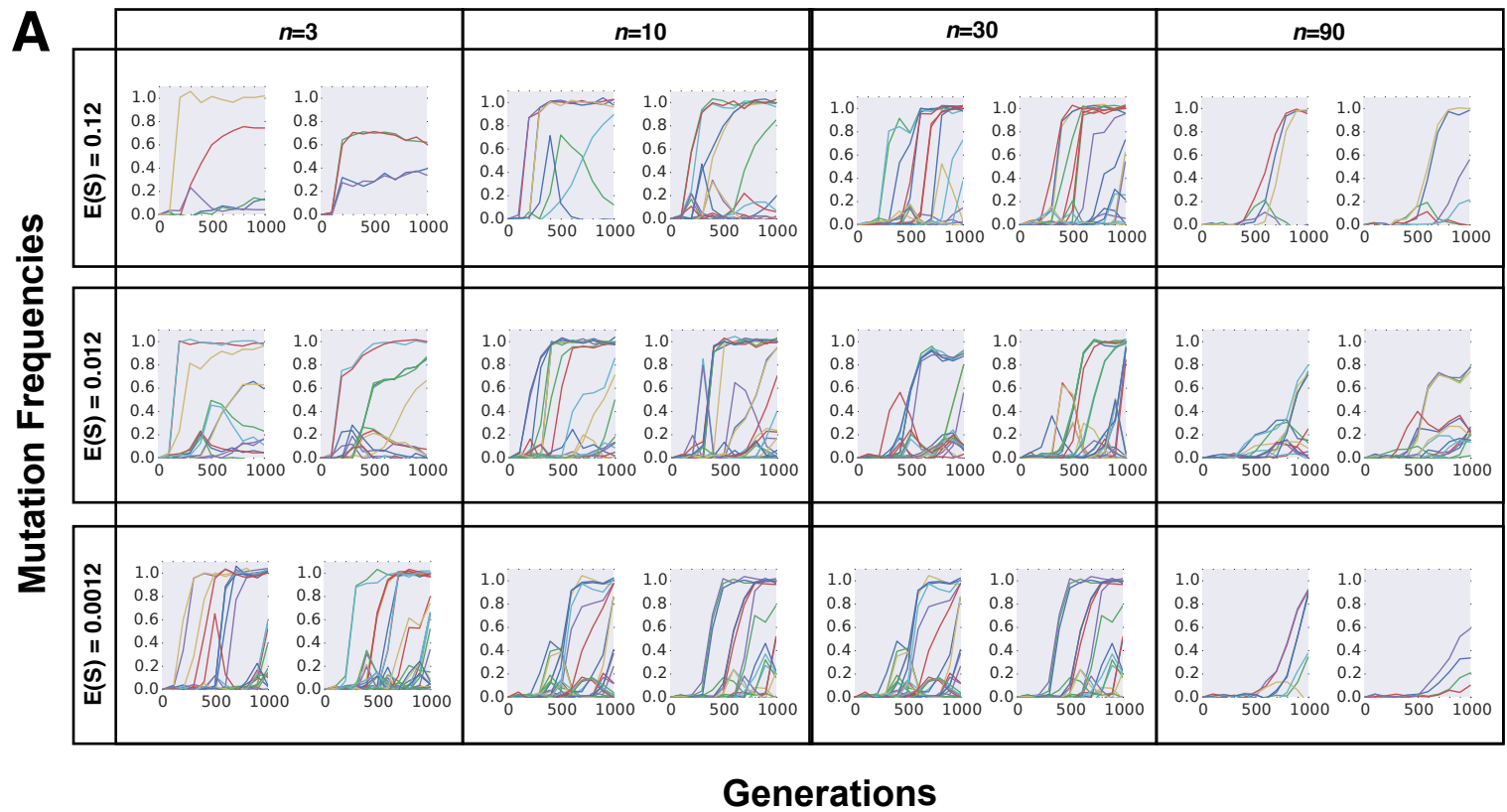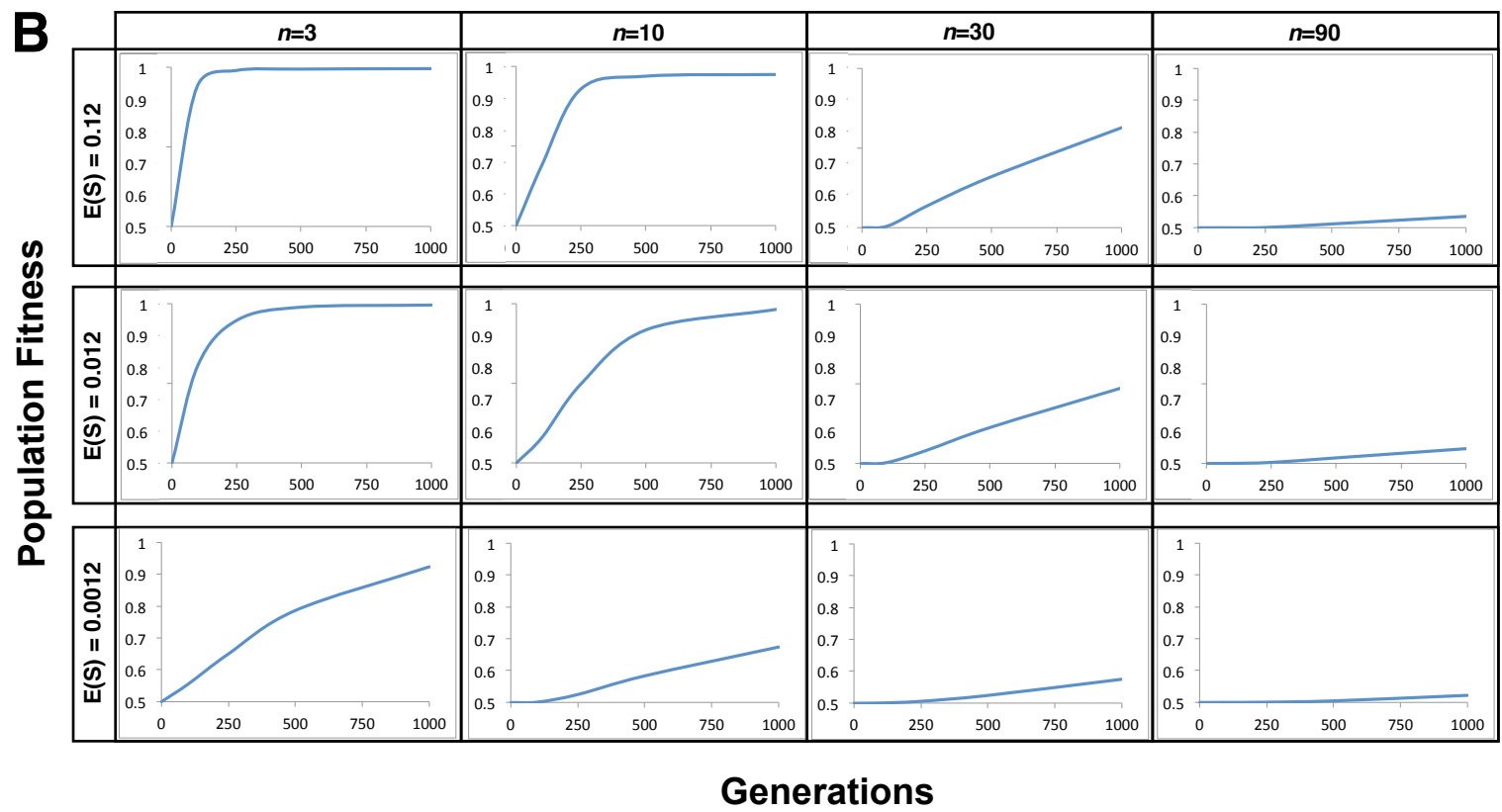

Supplement: Figure S5 — (A) Two representative simulations for each combination of parameters (all other parameters are as in Fig. 4): complexity (n) increases from left (n = 3) to right (n = 90), with a fixed m = 3; mean effect of mutation E(S) decreases from top (E(S) = − 0.012) to bottom (E(S) = − 0.0012). (B) Fitness increase for one of the two representative populations of each panel, for the same combination of parameters as shown in A. [file peerj-04-2256-s005.pdf]

Figure S6

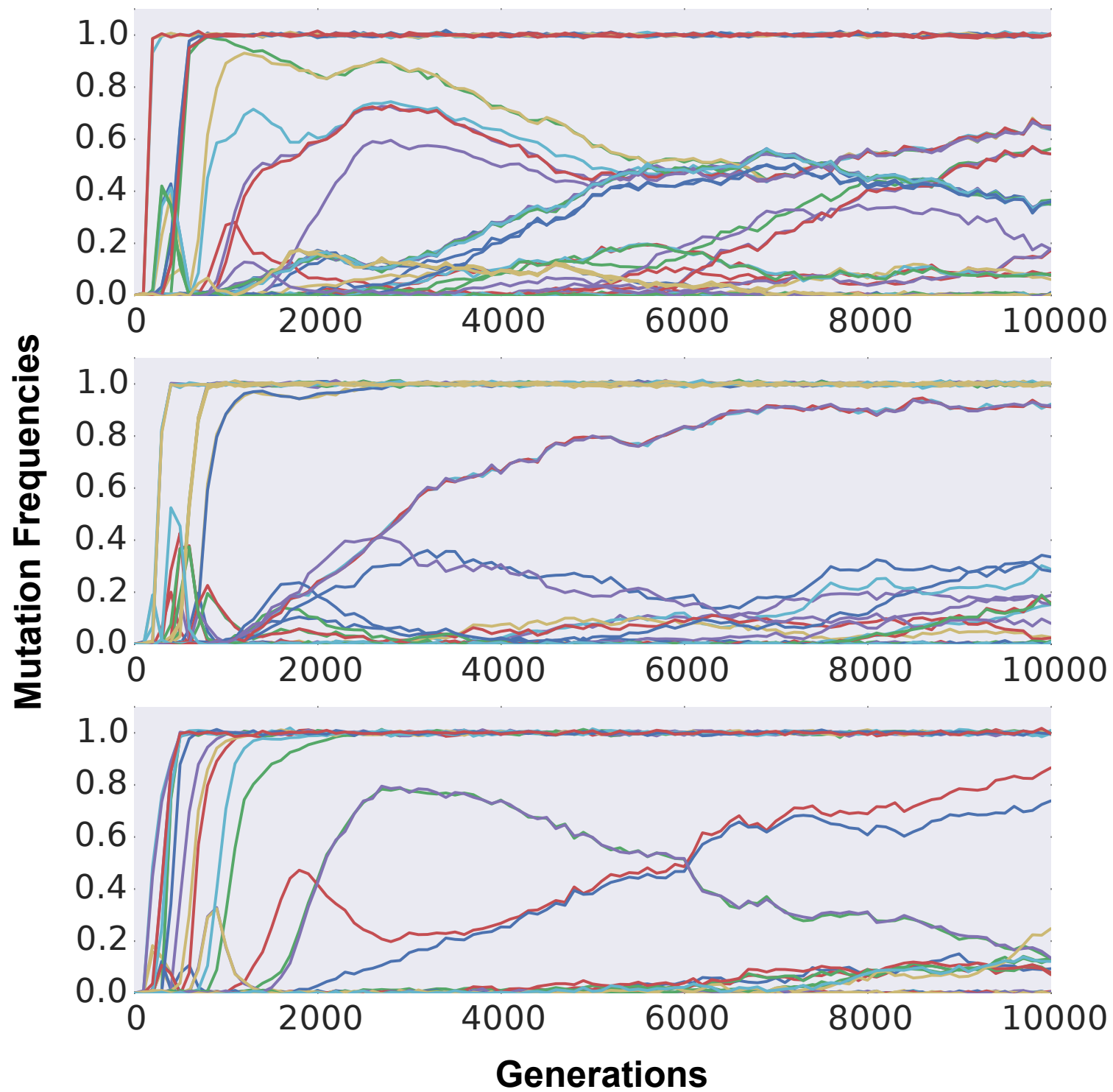

Supplement: Figure S6 — Three representative dynamics of the patterns of cohorts observed during 10,000 generations, indicating that under FGM polymorphisms can be maintained in populations for large periods of time. Parameters used are as in Fig. 4. [file peerj-04-2256-s006.pdf]

Figure S7

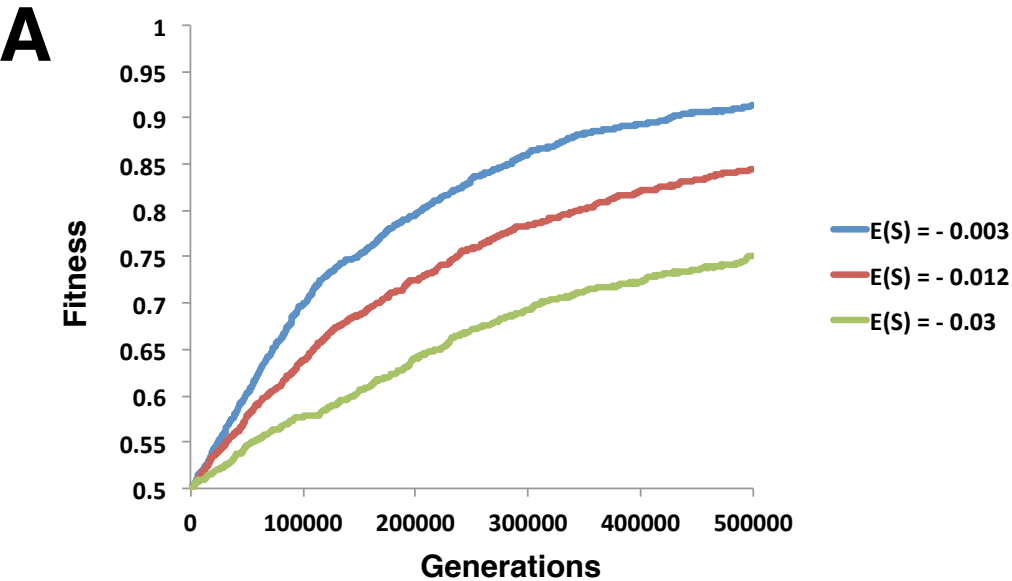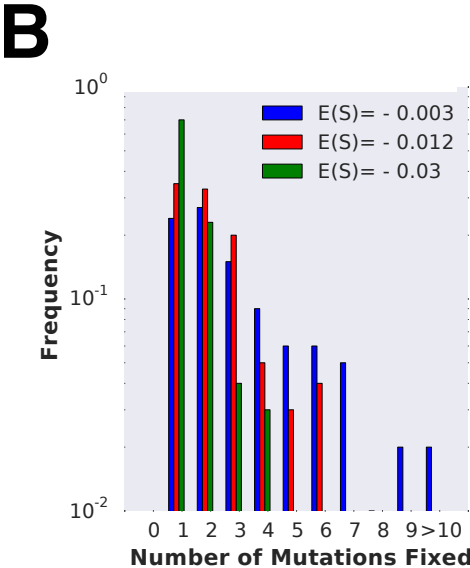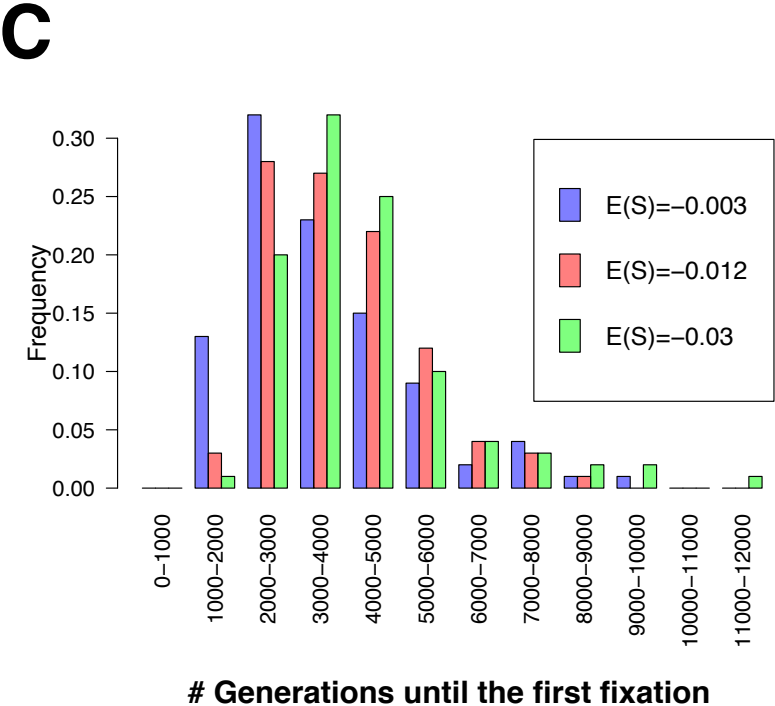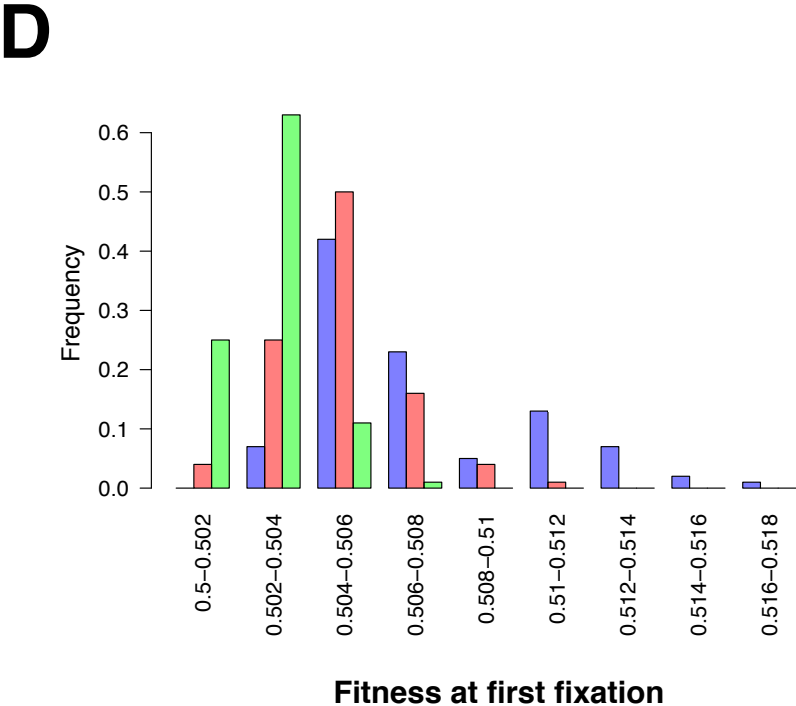

Supplement: Figure S7 — Simulations of adapting populations with high complexity (n = 500) and low pleiotropy (m = 3). Other parameters are: population size N = 104, initial fitness w0 = 0.5 and mutation rate U = 0.001. (A) Dynamics of fitness increase for populations with varying mean effect of mutations. (B) Probability distribution of the number of mutations fixed during the first fixation event (i.e., mutation cohort size). (C) Distribution of the number of generations until the first fixation. (D) Distribution of average fitness of populations at the first fixation event. In A, a single, representative simulation was used to assess the dynamics. B, C and D are the summary of 100 replicate simulations. [file peerj-04-2256-s007.pdf]
